# Finite Element Analysis of Stress and Displacement Patterns in the Maxillary Dentition During Miniscrew-Assisted en-Masse Retraction Using Variable Gable Bend Angles

**DOI:** 10.7759/cureus.86779

**Published:** 2025-06-26

**Authors:** Amit Maheshwari, Rushikesh Sonwane, Veerendra Kerudi, Anama Mahevi, Jay Patil, Mahavir Kotecha, Seema Gupta

**Affiliations:** 1 Department of Orthodontics, Jawahar Medical Foundation's (JMF's) Annasaheb Chudaman Patil Memorial (ACPM) Dental College, Dhule, IND; 2 Department of Orthodontics, Kothiwal Dental College and Research Center, Moradabad, IND

**Keywords:** bends, finite element analysis, maxilla, orthodontics, stress

## Abstract

Introduction: Efficient anterior tooth retraction with simultaneous intrusion is critical for the orthodontic treatment of deep bites. Gable bends incorporated into archwires can influence the direction and magnitude of force application. This study aimed to evaluate the effect of different gable bend angles (0°, 10°, 15°, and 20°) on stress distribution and displacement patterns in the maxillary dentition using three-dimensional (3D) finite element analysis (FEA) in the context of miniscrew-assisted en-masse retraction.

Methodology: A 3D geometric model of the adult maxilla was constructed using high-resolution computed tomography (CT) scan data. The maxilla was segmented using MIMICS 8.11 (Materialise NV, Leuven, Belgium), and surface refinement was performed using RapidForm (INUS Technology Inc., 3D Systems, Rock Hill, SC). The model was meshed in HyperMesh 13.0 (Altair Engineering, Inc., Troy, MI), and analysis was performed using ANSYS 12.1 (ANSYS, Inc., Canonsburg, PA). The model included teeth from the central incisor to the second molar, with a 0.25-mm-thick periodontal ligament (PDL) and alveolar bone. Orthodontic appliances included 0.022" McLaughlin, Bennett, and Trevisi (MBT) brackets (3M Unitek, Monrovia, CA) from central incisor to second premolar, bands on molars, a 0.019" x 0.025" stainless steel archwire (Ortho Organizers, Carlsbad, CA), and a transpalatal arch using 0.9 mm wire (GAC Int., Bohemia, NY). A miniscrew (1.5 mm × 10 mm; S.K. Surgicals, Pune, Maharashtra, India) was placed between the second premolar and first molar at an angle of 30°from the occlusal plane. A 200 g retraction force was applied using elastomeric chains (American Orthodontics, Sheboygan, WI) from a 6-mm crimpable hook to the miniscrew. Four models were created with gable bend angles of 0°, 10°, 15°, and 20°, placed 2 mm away from the canine bracket. Each model contained 82,566 nodes and 392,108 elements.

Results: The von Mises stress increased with greater gable bend angles, especially around the implant site and canine PDL. Cortical bone stress ranged from 51.9 MPa (0°) to 64.9 MPa (20°). The anterior teeth showed enhanced retraction and intrusion with 15° and 20° gable bends. The posterior teeth exhibited minimal displacement and maintained their anchorage. Intrusion and controlled tipping of the anterior teeth were more prominent at higher gable bend angles.

Conclusions: Gable bend angles of 15° and 20° provided optimal biomechanical conditions for en-masse retraction with effective intrusion of the maxillary anterior teeth while preserving the posterior anchorage. These configurations may offer clinical advantages for managing deep-bite cases.

## Introduction

Dental proclination, characterized by dento-alveolar flaring of maxillary or both maxillary and mandibular anterior teeth, is a prevalent malocclusion that often manifests as labially inclined incisors, increased overjet, and variable overbite, ranging from deep bite to open bite, leading to lip protrusion and facial convexity [1,2]. Such malocclusions pose significant aesthetic and functional challenges, making their correction a cornerstone of orthodontic treatment [2]. The primary objectives are to achieve an ideal overjet and overbite, align incisors, optimize torque and tip, ensure adequate arch length, and establish a Class I molar relationship [3]. These goals necessitate precise diagnosis, meticulous treatment planning, and efficient appliance design to simultaneously intrude and retract the anterior teeth while maintaining maximum anchorage, often following premolar extractions to reduce protrusion and enhance facial aesthetics [3].

Orthodontic intrusion and retraction are critical movements for correcting deep bites and proclined incisors [4]. Effective anchorage control is paramount to prevent unwanted tooth movements such as anchorage loss, which can compromise treatment outcomes [5]. Traditional anchorage reinforcement methods, such as transpalatal arches or headgears, often fail to prevent posterior segment movement, particularly in cases requiring significant anterior retraction [5]. The advent of temporary anchorage devices (TADs), notably miniscrews, has revolutionized orthodontic practice by providing direct skeletal anchorage, virtually eliminating anchorage loss, and reducing reliance on patient compliance [5]. Miniscrews are biocompatible, easy to insert and remove, cost-effective, and can be loaded immediately, making them ideal for en-masse retraction [5].

In sliding mechanics, biomechanical challenges, such as uncontrolled tipping or anchorage loss, are common because of unbalanced force systems [6]. To counter this, gable bends, which are angular deflections in the arch wire distal to the canine, are incorporated to generate moments that control tooth movement, prevent undesirable root apex displacement, and facilitate simultaneous intrusion and retraction [6]. The degree of gable bends significantly influences the moment-to-force (M/F) ratio, affecting the efficacy of en-masse retraction and intrusion, particularly in cases of deep bites [7]. However, the optimal degree of gable bends for specific treatment objectives is yet to be determined.

Finite element analysis (FEA), a non-invasive computational method, has emerged as a powerful tool for studying stress and displacement in orthodontic biomechanics [8]. By dividing complex structures, such as the maxilla, into finite elements, FEA enables precise evaluation of stress distribution, tooth movement, and periodontal ligament (PDL) behavior under varying conditions. This study employed FEA to investigate and compare the stress distribution and displacement patterns around the maxillary dentition during miniscrew-assisted en-masse retraction, utilizing varying degrees of gable bend, to identify the optimal configuration for effective intrusion and retraction of the anterior teeth during orthodontic treatment. The objectives included analyzing stress in the maxillary dentition, PDL, and alveolar bone under different gable bend angles and quantifying anterior tooth displacement to assess retraction and intrusion extents.

## Materials and methods

This in vitro study employed three-dimensional (3D) FEA to investigate the stress distribution and displacement patterns around the maxillary dentition during miniscrew-assisted en-masse retraction with gable bend angles of 0°, 10°, 15°, and 20°.

Hardware and imaging equipment

The study was conducted on a workstation computer equipped with an Intel Core 2 Duo processor operating at 2.1 GHz, 2 GB RAM, a 2 GB graphics card, a 320 GB hard disk, and a 17-inch monitor (Intel Corp., Santa Clara, CA). High-resolution Digital Imaging and Communications in Medicine (DICOM) data of the maxilla were acquired from a computed tomography (CT) scan of a patient to provide an anatomical basis for the model. Since, CT scan was acquired from the database with prior permission of the patient to use his record for research purposes, the ethical approval was not required for the study.

Software tools

The modeling and analysis processes utilized several specialized software tools. MIMICS 8.11 (Materialise NV, Leuven, Belgium) was used for visualization and segmentation of CT images to convert the DICOM data into a geometric model. ANSYS 12.1 (ANSYS, Inc., Canonsburg, PA) facilitated the FEA to simulate the stress and displacement. HyperMesh 13.0 (Altair Engineering, Inc., Troy, MI) was employed to convert the geometric model into an FEA model through meshing. RapidForm (INUS Technology Inc., 3D Systems, Rock Hill, SC) converted cloud data points into surfaces, which were exported in the Initial Graphics Exchange Specification (IGES) format.

Construction of the geometric model

A 3D geometric model of the adult human maxilla was constructed to represent the biological properties of the teeth, PDL, and alveolar bone. CT scan data of a human skull were processed using MIMICS 8.11 to extract the maxilla as the region of interest, converting DICOM data into a stereolithography (STL) format model comprising points, lines, surfaces, and volumes. The STL data were imported into RapidForm to generate surfaces, which were exported in the IGES format and further refined using HyperMesh 13.0. The PDL was modeled with an average thickness of 0.25 mm around the roots of all teeth to reflect anatomical variability. The model included teeth from the central incisor (CI) to the second molar bilaterally, equipped with McLaughlin, Bennett, and Trevisi (MBT) 0.022” brackets (3M Unitek, Monrovia, CA), bonded to the teeth from the central incisor to the second premolar, while the first and second molars were banded. A transpalatal arch, constructed with 0.9 mm (0.0354″) stainless steel (SS) wire (GAC Int., Bohemia, NY), connected the first molars palatally. A 0.019” x 0.025” SS arch wire (Ortho Organizers, Carlsbad, CA) was placed in the bracket slots. A titanium miniscrew, 1.5 mm in diameter and 10 mm in length (S.K. Chem Surgicals, Pune, Maharashtra, India), was positioned between the roots of the second premolar and first molar at the mucogingival junction, 12 mm from the archwire, at an angle of 30 degrees from the occlusal plane. An elastomeric chain (American Orthodontics, Sheboygan, WI) was used to apply a 200 g constant retraction force from a 6-mm anterior retraction hook (ARH) distal to the lateral incisor (LI) to the miniscrew.

FEA model generation

**Figure 1:**
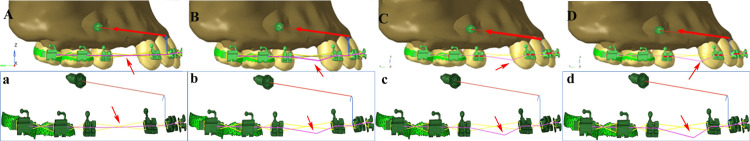
Finite element analysis (FEA) models. (A) Model 1 representing gable bend of 0°, (B) Model 2 representing gable bend of 10°, (C) Model 3 representing gable bend of 20°, (D) Model 4 representing gable bend of 30°. FEA models showing wire, bracket, and miniscrew assembly with 200 g of force application in model 1 (a), model 2 (b), model 3 (c), and model 4 (d).

The geometric model was converted into an FEA model using HyperMesh 13.0. Discretization was performed to represent the geometry with a finite number of elements and nodes, thereby enhancing the accuracy of the results. Tetrahedron elements were selected to mesh the irregular geometries of the teeth, PDL, and bone, thereby optimizing the computational efficiency of dental applications. Four models were created, each with a different gable bend angle in the SS arch wire distal to the canine (5 mm from the canine bracket): 0° (Model 1), 10° (Model 2), 15° (Model 3), and 20° (Model 4). Each model comprised 82,566 nodes and 392,108 elements, ensuring consistency across the simulations. The FEA model included the teeth, PDL, alveolar bone, brackets, arch wire, crimpable hook, and miniscrew (Figure 1).

Mesh convergence analysis

Mesh convergence analysis was conducted to ensure the accuracy and reliability of the FEA models. The mesh density was iteratively refined by increasing the number of elements from coarse to fine meshes while monitoring key output parameters such as maximum stress and displacement in the maxillary dentition. Convergence was achieved when the percentage change in the stress and displacement values between successive mesh refinements was less than 5%, indicating that further refinement yielded negligible improvements in accuracy. The final mesh, consisting of 82,566 nodes and 392,108 elements, was selected to balance computational efficiency with precision, accommodating the complex geometry and material properties of the maxilla.

Material property

The materials in the model, including cancellous bone, cortical bone, PDL, teeth, brackets, arch wire, crimpable hook, and miniscrew, were assumed to be homogeneous, isotropic, and linearly elastic. Material properties, such as Young’s modulus and Poisson’s ratio, were assigned based on values from a previous study to ensure anatomical accuracy and reflect the biomechanical behavior of each structure under applied forces (Table 1) [7].

**Table 1:** Material properties. MPa, megapascals

Material	Young’s modulus (MPa)	Poisson’s ratio
Tooth	20,000	0.31
Periodontal ligament	500	0.49
Cancellous bone	1,370	0.30
Cortical bone	13,700	0.30
Bracket	200,000	0.30
Arch wire/Hook	200,000	0.30
Stainless steel	200,000	0.30
Titanium	110,000	0.33

Boundary conditions

Boundary conditions were defined to prevent free-body motion and to simulate realistic constraints. The nodes on the outer surface of the maxillary bone were fixed in all directions, and the base of the maxilla was set as a fixed boundary condition for all models, ensuring stability during force application and the accurate simulation of biomechanical responses.

Loading configuration

A constant retraction force of 200 g was applied bilaterally using elastomeric chains from the miniscrew to the 6-mm ARH distal to the LI. The force was simulated in three axes: X (mesio-distal), Y (buccal-lingual), and Z (vertical), using ANSYS 12.1. The models were analyzed for movements of the crown and root of the maxillary CI, LI, canine, second premolar, first molar, and second molar, focusing on tipping and bodily distal movement. Von Mises stresses ( MPa) were calculated and presented in colorful contour bands, with different colors representing varying stress levels in the deformed state and positive or negative values indicating the direction of movement.

Interpretation of results

FEA was performed using ANSYS 12.1, and the results were post-processed to extract stress and displacement data. The movements of bones and stresses are presented as color bands, each representing different magnitudes. The displacements were calculated along the X-, Y-, and Z-axes to assess the biomechanical effects of the gable bend angles. The coordinate system for analyzing tooth movement during miniscrew-assisted en-masse retraction was defined as follows: for posterior teeth, a positive X-axis (+X) indicated distal movement, whereas a negative X-axis (-X) denoted mesial movement; for anterior teeth, a positive X-axis (+X) represented distal movement (retraction), whereas a negative X-axis (-X) indicated mesial movement (proclination). Regarding the Y-axis, for anterior teeth, a positive Y-axis (+Y) signified lingual crown movement (tipping), and a negative Y-axis (-Y) indicated lingual root movement, whereas for posterior teeth, a positive Y-axis (+Y) denoted lingual movement, and a negative Y-axis (-Y) represented buccal movement. On the Z-axis, a positive Z-axis (+Z) corresponded to intrusion and a negative Z-axis (-Z) corresponded to extrusion. This coordinate framework was used to interpret the stress and displacement patterns in the maxillary dentition across all models.

## Results

FEA was conducted to evaluate stress distribution during miniscrew-assisted en-masse retraction with varying gable bend angles (0°, 10°, 15°, and 20°) and revealed distinct patterns of von Mises stress across the cortical bone, cancellous bone, and PDL (Table 2).

**Table 2:** von Mises stress (MPa) in different structures of maxillary dentition in all the models. MPa, megapascals

Components	0°	10°	15°	20°
Cancellous bone	2.76	3.03	3.31	3.45
Cortical bone	51.94	57.13	62.33	64.92
Periodontal ligament	6.57 x 10^-5^	7.23 x 10^-5^	7.89 x 10^-5^	9.86 x 10^-5^

**Figure 2:**
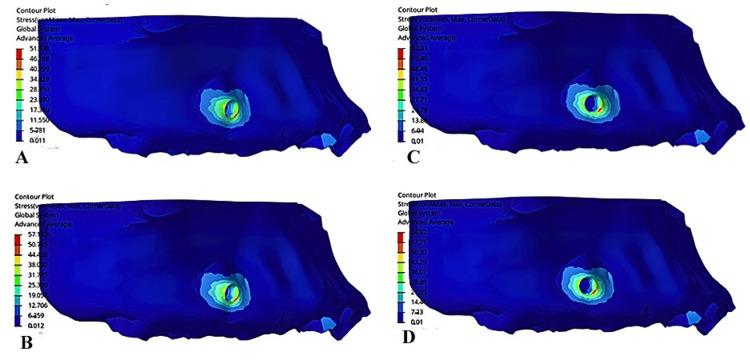
von Mises stress in cortical bone (in megapascals): (A) Model 1 (gable bend of 0°), (B) Model 2 (gable bend of 10°), (C) Model 3 (gable bend of 20°), (D) Model 4 (gable bend of 30°).

In cortical bone, the maximum von Mises stress was consistently observed at the implant fixation region, increasing progressively with the gable bend angle: 51.9 MPa at 0^0^, 57.13 MPa at 10^0^, 62.33 MPa at 15^0^, and 64.9 MPa at 20^0^. This trend indicated that higher gable bend angles amplified the stress concentration at the miniscrew site, likely owing to increased M/F ratios that alter the biomechanical load distribution (Figure 2).

**Figure 3:**
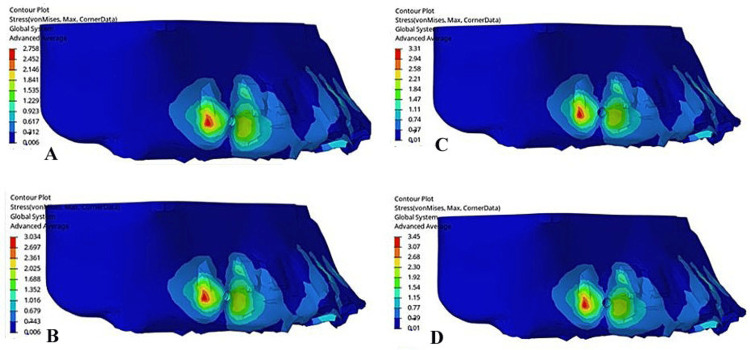
von Mises stress in cancellous bone (in megapascals): (A) Model 1 (gable bend of 0°), (B) Model 2 (gable bend of 10°), (C) Model 3 (gable bend of 20°), (D) Model 4 (gable bend of 30°).

In cancellous bone, the maximum von Mises stress, also at the implant fixation region, followed a similar pattern but at lower magnitudes: 2.7 MPa at 0°, 3.03 MPa at 10°, 3.31 MPa at 15°, and 3.45 MPa at 20°. The lower stress values in cancellous bone reflected its less dense structure compared to cortical bone; however, the incremental increase with the gable bend angle suggested heightened force transmission through the bone (Figure 3).

**Figure 4:**
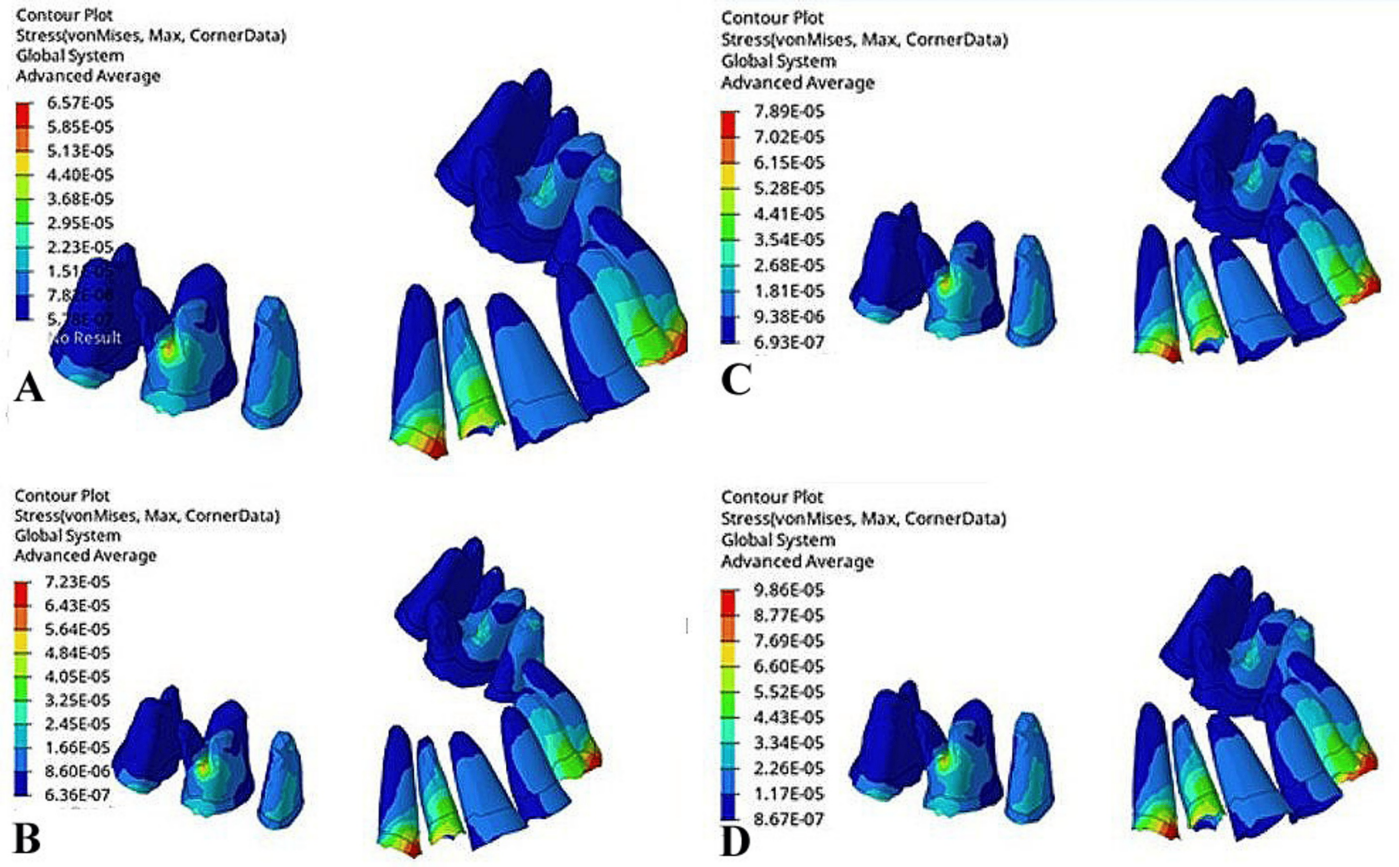
von Mises stress in the periodontal ligament (in megapascals): (A) Model 1 (gable bend of 0°), (B) Model 2 (gable bend of 10°), (C) Model 3 (gable bend of 20°), (D) Model 4 (gable bend of 30°).

In the PDL, the maximum von Mises stress was observed in the cervical region of the canine PDL, with values of 6.57 x 10^-5^MPa at 0°, 7.23 x 10^-5^ MPa at 10°, 7.89 x 10^-5^ MPa at 15°, and 9.86 x 10^-5^ MPa at 20°. The significantly lower stress in the PDL and its increase with the gable bend angle highlighted the role of the PDL in stress dissipation, with higher angles inducing greater stress concentration at the canine, possibly owing to enhanced tipping or intrusion forces (Figure 4).

FEA evaluating tooth movement during miniscrew-assisted en-masse retraction with gable bend angles of 0°, 10°, 15°, and 20° revealed distinct displacement patterns across the maxillary dentition in the mesio-distal (X), buccolingual (Y), and vertical (Z) directions. For the CI, at a 0° gable bend, minimal retraction was observed (X = 0.0001 mm), with slight lingual tipping movement (Y = 0.002 mm) and extrusion (Z = -0.002 mm), indicating limited retraction, undesirable tipping, and extrusion. At 10°, retraction increased (X = 0.001 mm) with more pronounced lingual crown movement (Y = 0.009 mm) and no vertical movement (Z = 0 mm), suggesting improved retraction control. At 15° and 20°, retraction remained consistent (X = 0.001 mm), but lingual crown movement decreased (Y = 0.007 and 0.005 mm, respectively), with intrusion increased (Z = 0.003 and 0.004 mm), indicating that higher gable bend angles facilitated both retraction and intrusion with controlled tooth movement, aligning with deep bite correction goals. The LI exhibited a similar trend. For posterior teeth, both second premolar and first molar showed mesial movement, which could have been due to retraction forces, and this movement was greater with a 20° gable bend. Moreover, the posterior teeth showed mild extrusive movements, which could have been due to the effect of gable bends (Table 3).

**Table 3:** Tooth movement (in mm) in the X, Y, and Z directions with gable bends at different angles. For posterior teeth (second premolar, first molar and second molar), a positive X-axis (+X) indicated distal movement, whereas a negative X-axis (-X) denoted mesial movement; for anterior teeth (central incisor, lateral incisor and canine), a positive X-axis (+X) represented distal movement (retraction), whereas a negative X-axis (-X) indicated mesial movement (proclination). Regarding the Y-axis, for anterior teeth, a positive Y-axis (+Y) signified lingual crown movement (tipping), and a negative Y-axis (-Y) indicated lingual root movement, whereas for posterior teeth, a positive Y-axis (+Y) denoted lingual movement, and a negative Y-axis (-Y) represented buccal movement. On the Z-axis, a positive Z-axis (+Z) corresponded to intrusion and a negative Z-axis (-Z) corresponded to extrusion.

Tooth	Axis	0°	10°	15°	20°
Central incisor	X	0.0001	0.001	0.001	0.001
	Y	0.002	0.009	0.007	0.005
	Z	-0.002	0	0.003	0.004
Lateral incisor	X	0.0001	0.001	0.002	0.002
	Y	0.002	0.010	0.007	0.005
	Z	-0.001	0	0.002	0.003
Canine	X	0.001	0.001	0.001	0.003
	Y	0.003	0.006	0.009	0.012
	Z	0	0	0.002	-0.001
Second premolar	X	-0.001	-0.001	-0.001	-0.002
	Y	0	0	0	0
	Z	0	0	-0.001	-0.001
First molar	X	-0.001	-0.001	-0.001	-0.002
	Y	0	0	0	0
	Z	0.001	0	-0.001	-0.002
Second molar	X	-0.002	-0.002	-0.002	-0.003
	Y	0	0	0	0
	Z	0.002	0	-0.002	-0.003

The canine showed consistent distal movement across all angles, starting at 0.001 mm at 0° and 10°, remaining stable at 15°, and increasing to 0.003 mm at 20°, indicating enhanced retraction at higher angles. Vertically, no movement occurred at 0° and 10° (Z = 0 mm), a slight intrusion appeared at 15° (Z = 0.002 mm), and minimal extrusion was noted at 20° (Z = -0.001 mm), implying variable vertical control. For the posterior teeth, the second premolar exhibited consistent mesial movement (X = -0.001 mm at 0°, 10°, and 15°, increasing to -0.002 mm at 20°, no buccal-lingual movement (Y = 0 mm across all angles), and minimal extrusion at 15° and 20° (Z = -0.001 mm), indicating anchorage stability at higher angles. The first molar showed similar mesial movement (X = -0.001 mm at 0°, 10°, and 15°; -0.002 mm at 20°), no buccal-lingual movement (Y = 0 mm), and variable vertical movement with slight intrusion at 0° (Z = 0.001 mm), no movement at 10° (Z = 0 mm), and extrusion at 15° and 20° (Z = -0.001 and -0.002 mm). The second molar displayed the greatest mesial movement (X = -0.002 mm at 0°, 10°, and 15°, -0.003 mm at 20°), no buccal-lingual movement (Y = 0 mm), and vertical shifts mirroring the first molar, with intrusion at 0° (Z = 0.002 mm), no movement at 10°(Z = 0 mm), and extrusion at 15° and 20° (Z = -0.002 and -0.003 mm). These findings suggested that the posterior teeth experienced mesial movement, with higher gable bend angles increasing the mesial and extrusive movements. Overall, the 15° and 20° gable bend angles optimized anterior retraction and intrusion of the anterior teeth, particularly the incisors, while maintaining acceptable anchorage control, making them more suitable for combined intrusion retraction and deep bite correction than 0° and 10°(Table 3).

## Discussion

The results of this study indicated that distal movement of the anterior teeth was noted in all models, with controlled tooth movement in the models where 15° and 20° gable bends were placed. This shows that TADs are highly effective for anchorage conservation in cases of en-masse anterior retraction. This finding was in agreement with previous studies where TADs have been used for en-masse retraction, providing controlled intrusion and retraction of the anterior teeth [5,9,10]. Kalia et al. [11] used FEA to evaluate miniscrew-assisted en-masse retraction and found that a high orthodontic mini-implant (10 mm) with a 6-mm ARH optimized controlled lingual tipping with minimal torque loss. Similar findings were reported by Kojima, Kawamura, and Fukui [12].

It was further revealed that the 15° and 20° gable bend configurations appeared most effective for achieving combined intrusion and retraction, particularly for the incisors, which showed consistent distal movement and intrusion. This is advantageous for deep bite correction, in which simultaneous vertical and sagittal control is critical. The 20° angle maximized canine retraction, but introduced a slight extrusion, suggesting that it may be less ideal for cases requiring pure intrusion. The 0° configuration, with minimal retraction and extrusion of the anterior teeth, was least suitable for en-masse retraction or deep bite correction, as it failed to deliver counter-tipping forces effectively. The 10° angle offered moderate retraction but lacked significant intrusion, limiting its utility for complex malocclusions.

Higher gable bend angles increase the M/F ratio, enhancing intrusion and retraction of the anterior teeth while minimizing root apex displacement, as evidenced by increased distal movement and intrusion at 15° and 20°​​​​​​​ in the central and lateral incisors, respectively. This biomechanical adjustment is used to achieve precise sagittal and vertical control, optimize anchorage, and facilitate efficient space closure in orthodontic treatment, particularly for Class I and Class II malocclusions requiring simultaneous intrusion and retraction [6]. Similar results were reported by Anh et al. [13], where an increased gable bend of 15°​​​​​​​ led to controlled tipping and intrusion of the anterior teeth.

However, these gable bends also led to mesial tipping and extrusion of posterior teeth, as observed in our study. The increased mesial and extrusive movement of posterior teeth during en-masse retraction with 15°​​​​​​​ and 20°​​​​​​​ gable bends was primarily due to the biomechanical interplay of forces and moments. The gable bends create a moment to counteract lingual tipping of the anterior teeth with counterclockwise moments [6,7], but the retraction force, applied at the 6-mm ARH (below the anterior segment’s center of resistance at ~8 to 10 mm) [14], still induces a slight clockwise rotation of the arch, causing mesial tipping of posterior teeth. Additionally, the gable bends introduce a vertical force component, transmitting an extrusive force to the posterior teeth via the continuous archwire, exacerbated by wire-slot play and friction in sliding mechanics. The mini-implant at 12 mm provided skeletal anchorage [11], but residual reciprocal forces and moments from the gable bends might have contributed to anchorage loss, leading to increased mesial and extrusive movement of the posterior teeth [6,7]. 

In the FEA study conducted by Sung et al. [14], it was observed that despite the application of the retraction force vector in proximity to the center of resistance (CR) for the six anterior teeth with an 8-mm ARH, neither the CI nor the LI underwent bodily retraction. The authors proposed that the incorporation of compensating curves (gable bends) and an auxiliary mini-implant positioned between the incisors to apply vertical forces would mitigate the constraints imposed by ARH. As indicated by the authors, the introduction of an additional mini-implant to exert midline vertical traction could potentially lead to an unnecessary increase in morbidity.

A research investigation conducted by Mo et al. [15] determined that a gable bend of 20°​​​​​​​ with an ARH of 8 mm resulted in a translational movement of the anterior dentition. An increase in the degree of the gable bend corresponded to a more significant retraction of the anterior teeth, a phenomenon that was likewise observed in our study. In the absence of a gable bend, the retraction force induces a clockwise moment as the force vector traverses beneath the CR of the six anterior teeth. This moment results in a reduction in the anterior torque and promotes extrusion of the anterior teeth [7]. A gable bend in the SS archwire segment produces a vertical force. As an ARH was situated between the LI and the canine, the vertical force vector was directed anterior to the anterior segment of the CR. Consequently, this vertical force induced a counterclockwise moment that governed the movement of the anterior teeth during the en-masse retraction. An increase in the gable bend enhances both the vertical force and counterclockwise moment [6,7].

The trends in our study suggest that higher gable bend angles amplified the M/F ratio, intensifying stress transmission through the bone and PDL, particularly at the canine, because of its proximity to the gable bend. The stress levels in the cortical bone were within safe limits for bone remodeling (typically <100 MPa) [16]; however, the progressive increase raised concerns about miniscrew stability at 20°, where the stress approached critical thresholds. The lower stress in the cancellous bone and PDL indicated their roles in stress disorders, yet the increase in stress in the canine PDL suggests the potential for localized strain, which could influence tooth movement dynamics. These findings are consistent with those of a previous study [17,18], where increased M/F ratios in sliding mechanics enhanced the force distribution, but increased the risk of anchorage unit stress.

The findings from this FEA study on miniscrew-assisted en-masse retraction with varying gable bend angles offer valuable clinical guidance for orthodontists treating Class I and Class II malocclusions, particularly those with deep bites and proclined anterior teeth. Higher gable bend angles, specifically 15°​​​​​​​ and 20°​​​​​​​, were found to be the most effective for achieving combined intrusion and retraction of the anterior teeth, making them ideal for deep bite correction, where both vertical and sagittal control are critical for aesthetic and functional outcomes. The 15°​​​​​​​ angle provided a balanced approach, minimizing undesirable posterior tooth extrusion, whereas the 20°​​​​​​​ angle, although effective, introduced slight canine extrusion, which could complicate severe cases. Lower angles, such as 0°​​​​​​​ and 10°​​​​​​​, were less suitable because they resulted in minimal retraction and failed to achieve significant intrusion, potentially worsening the vertical discrepancies. The observed increase in stress in the cortical bone and PDL at higher angles underscores the need for precise miniscrew placement and regular monitoring to ensure bone and screw stability. These insights enable orthodontists to optimize appliance design, tailor gable bend selection to specific treatment goals, and enhance efficiency and predictability in complex orthodontic cases.

This study had several limitations. Its in vitro nature assumes homogeneous and isotropic material properties for the bone and PDL, potentially oversimplifying their anisotropic behavior in vivo, which might affect stress and displacement accuracy. The fixed retraction force and ARH height may not reflect the clinical variability, thus limiting the generalizability of our findings. Our model focused solely on the maxillary arch, excluding mandibular interactions that could influence Class II malocclusions. Long-term bone remodeling and PDL adaptation, which are critical for clinical outcomes, have not yet been simulated. Additionally, the reliance of this study on a single maxillary model may not account for patient-specific anatomical variations. Future in vivo studies incorporating dynamic loading, patient-specific models via cone-beam CT, and a broader range of gable bend angles are required to validate and extend these findings.

## Conclusions

This FEA study on miniscrew-assisted en-masse retraction demonstrated that higher gable bend angles, particularly 15^°^ and 20^°^, optimized the combined intrusion and retraction of the anterior teeth. The 15^°^ angle balanced retraction and intrusion with minimal posterior anchorage loss, whereas the 20^°^ angle, although effective, resulted in slight canine extrusion. Lower angles (0^°^ and 10^°^) were inadequate to achieve significant retraction or intrusion. Increased stress in the cortical bone and PDL at higher angles necessitates careful mini-screw placement. These findings will guide orthodontists in selecting gable bend configurations to enhance treatment precision and outcomes.
